# Reply to the critics on “binge drinking and alcohol prices”

**DOI:** 10.1186/s13561-016-0084-8

**Published:** 2016-02-02

**Authors:** Jon P. Nelson

**Affiliations:** Department of Economics, Pennsylvania State University, University Park, PA 16802 USA

## Introduction

Xuan et al. [1] offer several “interpretive” comments on my systematic review [2] of binge drinking and alcohol prices, including comments concerning selection and interpretation of primary studies, alternative methodologies, and supporting literatures. Prior to addressing these issues, it is important to layout what has transpired in the addiction field as reflected in the editorial policies of the academic journal, *Addiction*, for which Professor Babor was Associate Editor-in-Chief. This provides context to faulty comments made by Xuan et al. [1]. Starting in 2009 or earlier, *Addiction* has engaged in a campaign against publication of any academic alcohol research that received industry support or sponsorship [3–6]. As stated by Stenius and Babor ([6], p. 191), “. . . all financial relationships with the alcoholic beverage industry are [best] avoided”. Censorship always begins with appeals to base emotions and feelings, so the campaign is surrounded with emotion-laden words that make for entertaining reading – such as “transparency,” “vested interests,” “gatekeepers,” and of course “biased findings.” Economists generally welcome open debate in the marketplace for ideas regardless of source, including contributions from researchers who rely heavily on “non-profit” financial support. For my own part, I acknowledged support received from the International Center for Alcohol Policies ([2], p. 11). All of my publications supported by ICAP contain the same declaration, and I see no reason to go beyond a simple, direct statement. Other related work that has been dormant for a decade is irrelevant and -- as I work independently as well -- did not result in research publications. That some of my conclusions do not coincide with those of Xuan et al. is to be expected in any area of scientific research. Scientific inquiry should be associated with a diversity of ideas, methods, and results, and not the monolithic approach advocated by Babor and associates.

## Interpretation and selection of several primary studies

My systematic review of binge drinking [2] examines 56 econometric studies, five natural experiments, and six field studies. A qualitative approach was chosen, since primary studies report findings for numerous heterogeneous outcomes and employ different data sets and statistical methods. An on-line Supplemental File provides more details on each primary study, including summary comments on conflicting results obtained with different model specifications. It is unclear if the critics examined this File.

Qualitative reviews are assessments and not mere summaries; hence reviewers do not always agree with primary authors’ interpretation or statement of results; see [7] for extended examples. Xuan et al. [1] object to my summary of a study, Xuan et al. [8], containing “diverse” results for youth binge drinking. My tabular summary indicates insignificant results for taxes when the adult binge rate is included as a variable in the regression model ([2], p. 7), and I report conflicting results in the Supplemental File. Xuan et al. [1, 8] emphasize an indirect or mediated result through adult binging, whereas my review (and all other primary studies in my review) focus on direct or primary effects of taxes on youth drinking. The basis for my assessment was the following statement:

*However, after adjustment for adult binge drinking, the association between tax and youth drinking was attenuated and no longer statistically significant (AOR = 0.98, 95 % CI: 0.90, 1.07). We observed similar findings when assessing the effect of adult binge drinking on the relationship between tax and youth binge drinking (*[8]*, p. 1717).*

In other words, taxes have an insignificant direct effect on youth bingeing, but the authors argue that “alcohol taxes may affect youth drinking through their effects on adult drinking . . . [but] causal inference is limited” ([8], p. 1718). Insignificant direct results may be counter-intuitive, at least given the emphasis in the addiction literature on alcohol price responses by youth (e.g., [9, 10]). Further, indirect effects inevitably are small in magnitude as a policy basis. Xuan et al. may have found a correlation, but their explanation for causation is not proven. When causal inference is limited, then several explanations are possible, but they [1, 8] offer only one. My interpretation of their results is supported by 14 of the remaining 17 studies for youth. Two other econometric studies [11, 12] also examine indirect effects and report insignificant direct relationships between taxes and youth or young adult drinking, but neither of these studies is cited in [8]. The results in [8] are not strong evidence in favor of a particular interpretation of statistical relationships.

Two other objections are raised by Xuan et al. [1] to studies that I cited (or omitted). These can be dealt with in summary fashion. First, Nelson et al. [13] was included as an example of a study that “draw[s] a general policy link between alcohol prices and excessive alcohol consumption” ([2], p. 2), and was cited along with prior prominent studies (e.g., [14]). Nelson et al. state that their Delphi study of alcohol policies “builds on prior work” ([13], p. 26) with regard to strength of policy evidence and by extension it reflects what is known or believed by public health researchers regarding aggregate econometric studies, such as the prior work I cited [14]. Second, my review summarizes empirical results in a 2001 publication by Cook and Moore [11], but their second article in 2002 [10] is a policy review that contains no new econometric results. All of the objections raised by Xuan et al. [1] regarding interpretation or selection of empirical results are groundless.

## Methodology: meta-analysis vs. qualitative reviews

I have written at length about the methodology of meta-analysis as applied to econometric studies [15, 16], and have used meta-analysis for a number of different data sets, including several meta-regression analyses of alcohol effects (e.g., [7, 17, 18]). Xuan et al. [1] ignore these critiques and analyses, including my past comments [17–19] that are highly critical of several works that they cite such as Wagenaar et al. [20]. Briefly, past meta-analyses of alcohol prices and alcohol-related harms are deeply flawed by incomplete data sets; lack of comparable quantitative measures; improper weighting of average effect-sizes; lack of controls for publication bias; lack of adjustments for non-independent observations; failure to properly employ meta-regression techniques; and other econometric problems. Further, results in [20] for “heavy drinking” are confined to only 10 studies and some results in Elder et al. [21] are based on even smaller sample sizes. Combining “apples and oranges” via a quantitative summary or using very small sample sizes are to be avoided. Qualitative reviews that are comprehensive are complements to quantitative meta-analyses, and I see no reason to view one approach as necessarily superior to the other, especially in light of methodological errors made in past meta-analyses. My statement of a quantitative standard for statistical significance was carefully explained in my review ([2], p. 5–6).

## Supporting studies: controlled experiments vs. natural experiments

It comes as a surprise that Xuan et al. [1] seek additional evidence from laboratory experiments, but cite only two old “controlled” experiments by Babor and associates that use the same small sample of 34 volunteer drinkers. No interrupted time-series (ARIMA) studies are cited or other natural experiments. However, Babor and associates state the following about advantages of different primary studies, data collections, and methodologies:

“Studies of what happens when there is a change – an implemented or discontinued intervention – provide the most valuable evidence on the effects of alcohol policy . . . the various modes of data collection have different advantages and drawbacks . . . [but] in general, as one moves from individual-level to population-level interventions, the utility of [controlled] experimental methods becomes problematic. One reason is that controlled experiments may not be the best way of evaluating behaviours and outcomes in complex real-life settings ([14], p. 105).

Xuan et al. [1] could have instead chosen to review natural or quasi-experiments, including those that connect alcohol prices to alcohol harms in real-life settings. Recently, I have completed a systematic review of 55 studies of natural experiments for interventions involving alcohol prices in four European countries and Hong Kong [Nelson JP. What happens to drinking and harms when alcohol policy changes? A systematic review of five natural experiments for alcohol taxes, prices, and availability. Unpublished SSRN Working Paper 2612580. 2015]. These studies contain 78 results for alcohol consumption or harms and alcohol taxes. A number of ARIMA studies also are included. Results for binge drinking in this review support my conclusions in [2] as well as more selective results in my other reviews in this area [22, 23]. This is important in light of the fact that a population-level approach to alcohol policy is favored by public health researchers, including my critics. Binge drinking is not a trivial public health problem, nor did I suggest that it was, but the critics do a disservice to other researchers and policymakers by pretending to be informed authorities on all counts in their faulty set of comments.
